# Phenotypic and biochemical characteristics and molecular basis in 36 Chinese patients with androgen receptor variants

**DOI:** 10.1186/s13023-021-01765-w

**Published:** 2021-03-09

**Authors:** Hui Zhu, Haijun Yao, Yue Xu, Yan Chen, Bing Han, Nan Wang, Hao Wang, Qiang Zhang, Wenjiao Zhu, Yuanping Shi, Hua Sun, Shuangxia Zhao, Huaidong Song, Yang Liu, Jie Qiao

**Affiliations:** 1 Department of Endocrinology, Shanghai Ninth People’s Hospital, Shanghai Jiao Tong University School of Medicine, Shanghai, 200011 China; 2 Department of Urology, Shanghai Ninth People’s Hospital, Shanghai Jiao Tong University School of Medicine, Shanghai, 200011 China; 3 Department of Obstetrics and Gynecology, Shanghai Ninth People’s Hospital, Shanghai Jiao Tong University School of Medicine, Shanghai, 200011 China; 4 Research Centre for Clinical Medicine, Shanghai Ninth People’s Hospital, Shanghai Jiao Tong University School of Medicine, Shanghai, 200011 China; 5 Department of Plastic Surgery, Shanghai Ninth People’s Hospital, Shanghai Jiao Tong University School of Medicine, Shanghai, 200011 China

**Keywords:** Androgen insensitive syndrome (AIS), Androgen receptor (AR) mutation, Disorder/differences of sex development (DSD), Functional assay

## Abstract

**Background:**

Androgen insensitive syndrome (AIS) is a rare genetic disease resulting from androgen receptor (*AR*) mutations and one of the causes of 46, XY disorder of sexual development (DSD). This study aimed to describe the clinical features and molecular defects of 36 Chinese patients with *AR* variants and investigate the functional alterations of novel variants in vitro.

**Material and methods:**

Subjects with *AR* variants were identified from 150 Chinese 46, XY DSD patients using targeted next-generation sequencing. In-silico and functional assays were performed to evaluate the transcriptional activity and nuclear localization of novel *AR* variants.

**Results:**

Eight novel and fifteen reported *AR* variants were identified. 30.6% (11/36) of patients harbored additional variants other than *AR*. Mutations in the Arg841 residue were found in 7 unrelated patients. Postpubertal serum gonadotropin levels were significantly elevated in patients with complete AIS (CAIS) compared with those in patients with partial AIS (PAIS) (*P* < 0.05). All the novel variants initially predicted to be uncertain significance by in-silico analyses were reclassified as likely pathogenic for defective AR transcriptional activity in vitro, except p.L295P, which was found in an atypical patient with oligogenic mutations and reclassified as likely benign. c.368_369 ins T was observed to interfere with nuclear translocation.

**Conclusions:**

Compared with PAIS patients, postpubertal CAIS patients had higher gonadotropin levels. Arg841 was disclosed as the location of recurrent mutations in Chinese AIS patients. Functional assays are important for reclassifying the novel *AR* variants and re-examining the diagnosis of AIS in specific patients with oligogenic mutations, instead of in-silico analysis.

**Supplementary Information:**

The online version contains supplementary material available at 10.1186/s13023-021-01765-w.

## Background

Androgen insensitivity syndrome (AIS, OMIM:300068) is the common cause of 46, XY disorders/differences of sex development (DSDs) [1–3], and is characterized by a wide phenotypic spectrum ranging from a total female appearance of the external genitalia to a nearly complete male phenotype with mild symptoms of gynecomastia [1]. This disorder is divided into three subtypes: complete AIS (CAIS), with the complete feminization of the external genitalia; partial AIS (PAIS), with a variable clinical presentation; and mild AIS (MAIS), with normal male external genitalia but gynecomastia and/or infertility in puberty or adulthood [2]. As a rare genetic disease, the incidence of AIS varies among different ethnic backgrounds and ranges between 1:20,000 and 1:99,000 in genetically 46, XY males [4].

The process of male sexual differentiation and maturation is strictly related to androgen and normal androgen receptor (AR) function [5]. AR is a nuclear receptor and allows target cells to respond to the biological action of androgen by binding testosterone (T) or dihydrotestosterone (DHT) [4], which is critical for the physiological virilization of male genitalia that occurs between the 8th and 14th weeks of gestation [6]. AIS is an X-linked recessive disorder caused by pathogenic mutations in the *AR* gene that lead to complete or partial resistance to the physiological effects of androgen in 46, XY individuals [7].

The *AR* gene (*NR3C4*) is located at chromosome Xq11-12 and consists of eight exons [8]. The entire receptor consists of three functional domains: the variable N-terminal domain (NTD), the conserved DNA-binding domain (DBD) and the conserved C-terminal ligand-binding domain (LBD) [9, 10]. To date, there are more than 800 reported mutations in the *AR* gene related to AIS [11], most of which were predicted to be pathogenic by in-silico analyses. However, these bioinformatic tools tend to show low specificity, resulting in the overprediction of missense alterations as deleterious. Therefore, it is not recommended that these in-silico predictions could be used as the sole source of evidence to make a clinical assertion [12].

DHT-dependent transcriptional induction of the androgen-regulated *APOD* (apolipoprotein D) gene in fibroblasts was used to evaluate the function of the androgen receptor [13], but the invasiveness limited the application, and it was not helpful to illustrate the functional defects of the variants when next-generation sequencing (NGS) was widely used in the molecular diagnosis. The present study described the clinical features and molecular basis of 36 Chinese 46, XY DSD patients with *AR* variants that were identified using targeted NGS and investigated the functional alterations of eight novel *AR* variants in vitro.

## Materials and methods

### Patients

150 Chinese 46, XY DSD patients, who visited our hospital from 2009 to 2019 and could not be diagnosed according to the typical clinical phenotypes and routine candidate gene strategies, were recruited for this study. The phenotypes of these patients ranged from different degrees of undermasculinization to a nearly complete female appearance. The Ethics Committee (Institutional Review Board) of the Ninth People’s Hospital of Shanghai approved all protocols. Patients provided written informed consent.

### Next-generation sequencing and data analysis

A total of 80 related genes were selected in the NGS panel (Additional file 1: Table S1) [3]. Genomic DNA of 150 patients was extracted from peripheral blood lymphocytes and genomic sequences were obtained from the University of California, Santa Cruz and National Center for Biotechnology Information databases. We completed the targeted exome design and capture by using the Access Array system and Illumina Sequencing System (Fluidigm, San Francisco, CA, USA). The entire coding regions and exon–intron boundaries of the genes were amplified by multiplex PCR on the Access Array™ microfluidics platform (Fluidigm, San Francisco, CA, USA) [3]. Variants with a frequency of < 1% were retained and ultimately confirmed by Sanger sequencing. Targeted, capture-based DNA sequencing was used in patient 36, who showed classical CAIS, but found no mutation by above-mentioned method.

### Assessment of variants

According to the guidelines published by the American College of Medical Genetics and Genomics (ACMG), the variants were classified into five categories: pathogenic, likely pathogenic, variants of uncertain clinical significance (VUS), likely benign and benign [12]. Novel *AR* variants were predicted to be damaging or not by in-silico analyses [3]. Because the well-established in vitro functional study is one of the strong levels for assessing the pathogenicity of variants (PS3 and BS3), all the novel *AR* variants were reclassified after in vitro functional assays [12].

### Clinical data

Thirty-six subjects with identified *AR* variants were enrolled for further study. The clinical data were collected at the subjects’ first visit to our hospital without any medication, however, patients 5 and 9 had already undergone ‘inguinal hernia surgery’. External masculinization score (EMS) was calculated to obtain a quantitative measure of the degree of undermasculinization [14]. Serum T, luteinizing hormone (LH) and follicle-stimulating hormone (FSH) levels were measured using chemiluminescent immunoassays (Abbott Diagnostics, Abbott Park, IL, USA), and serum anti-Mullerian hormone (AMH) was measured by electrochemiluminescence immunoassay (Roche, Switzerland). The androgen sensitivity index (ASI), which is frequently higher in individuals with AIS, was defined as the product of LH and T: ASI = LH (mIU/ml) × T (ng/ml) [15]. All the AIS patients were divided into CAIS and PAIS subgroups according to the clinical phenotype.

### Plasmid construction

pCMV-hAR-WT and pRL-SV40 were kindly provided by Dr. Shigeaki Kato (University of Tokyo, Tokyo, Japan). p(ARE)_4_-Luc, which contains four synthetic tandem repeats of an androgen-responsive element (ARE), was a kind gift from Dr. Xin Yuan from Beth Israel Deaconess Medical Center, Harvard Medical School. Wild-type (WT) human *AR* cDNA was amplified from pCMV-hAR by PCR using specific primers with restriction enzyme sites (Xho I-BamH I) and subsequently cloned into the expression vector pEGFP-N2 to generate pEGFP-N2-AR-WT (the primers are presented in Additional file 1: Table S2).

### Site‑directed mutagenesis of *AR*

Eight mutant AR expression vectors carrying the novel variants, including c.368_369 ins T, p.L295P, p.A588P, p.G590R, p.R761W, p.L812Q, p.F879C and p.I907V, were created by site-directed mutagenesis (TRANSGEN Biotech, China) using pCMV-hAR-WT and pEGFP-N2-AR-WT as the templates respectively. The primers used for mutagenesis are listed in Additional file 1: Table S2. Before further functional studies, the entire coding sequences of all the mutant plasmids were confirmed by Sanger sequencing.

### Transcriptional assays

Dimerized ARs translocate into the nucleus and bind to the ARE sequences found in various AR target genes to initiate a complex transcriptional program in target cells, such as LNCaP, the human prostate cancer cell line [16]. Transcriptional assays were performed in 24-well plates using Lipofectamine 2000 (1 μl/well) (Invitrogen, Waltham, MA, USA). Empty vector, WT and mutant AR expression vectors (200 ng/well) were cotransfected into LNCaP cells with p(ARE)_4_-Luc (200 ng/well) and pRL-SV40 (10 ng/well) containing Renilla luciferase as an internal indicator. After 24 h, the LNCaP cells were incubated in RPMI 1640 containing 10% dextran-coated charcoal-stripped fetal bovine serum and 10^–7^ M DHT.

To eliminate the possible influence of the LNCaP cell line or DHT concentration on the functional activity of mutant ARs with p.L295P, transcriptional assays were repeated under the same conditions but in the embryonic kidney (HEK) 293T cells incubated with Dulbecco's modified Eagle's medium containing 10% FBS containing 10^–7^ M, 10^–6^ M or 10^–5^ M DHT for 24 h.

LNCaP and 293T cells were washed twice with PBS and lysed via incubation with Passive Lysis Buffer (100 μl/well, Promega Co., Madison, WI) for 20 min. The lysate (20 μl) in each well was sampled to measure the firefly and Renilla luciferase activities using the Dual-Luciferase Reporter assay system (Promega, Madison, WI, USA). The results were normalized to the internal Renilla control and presented as the relative luciferase activity. All the experiments were carried out in triplicate wells and repeated three times.

### Subcellular localization

The pEGFP-N2 vector, pEGFP-N2-AR and 8 novel mutants were transfected into 293T cells in a confocal dish with Lipofectamine 2000 (5 μl). The cells were incubated with 10^–7^ M DHT except for one dish transfected with WT AR. The medium was removed after 24 h. The cells were washed three times with PBS and fixed with 4% paraformaldehyde for 15 min. Nuclear counterstaining was performed with Hoechst (Hoechst AG, Frankfurt, Germany) for 5 min, and images were obtained by confocal laser scanning microscopy (Nikon A1, Japan).

### Statistical analysis

Data are presented as medians with interquartile range. The Mann–Whitney U test was performed for comparisons of hormone levels between CAIS and PAIS patients and for AR functional assays using GraphPad Prism 8.0 software (GraphPad Software, San Diego, CA, USA). A two-sided *P* value < 0.05 indicated a significant difference.

## Results

### *AR* variant analysis

**Fig. 1:**
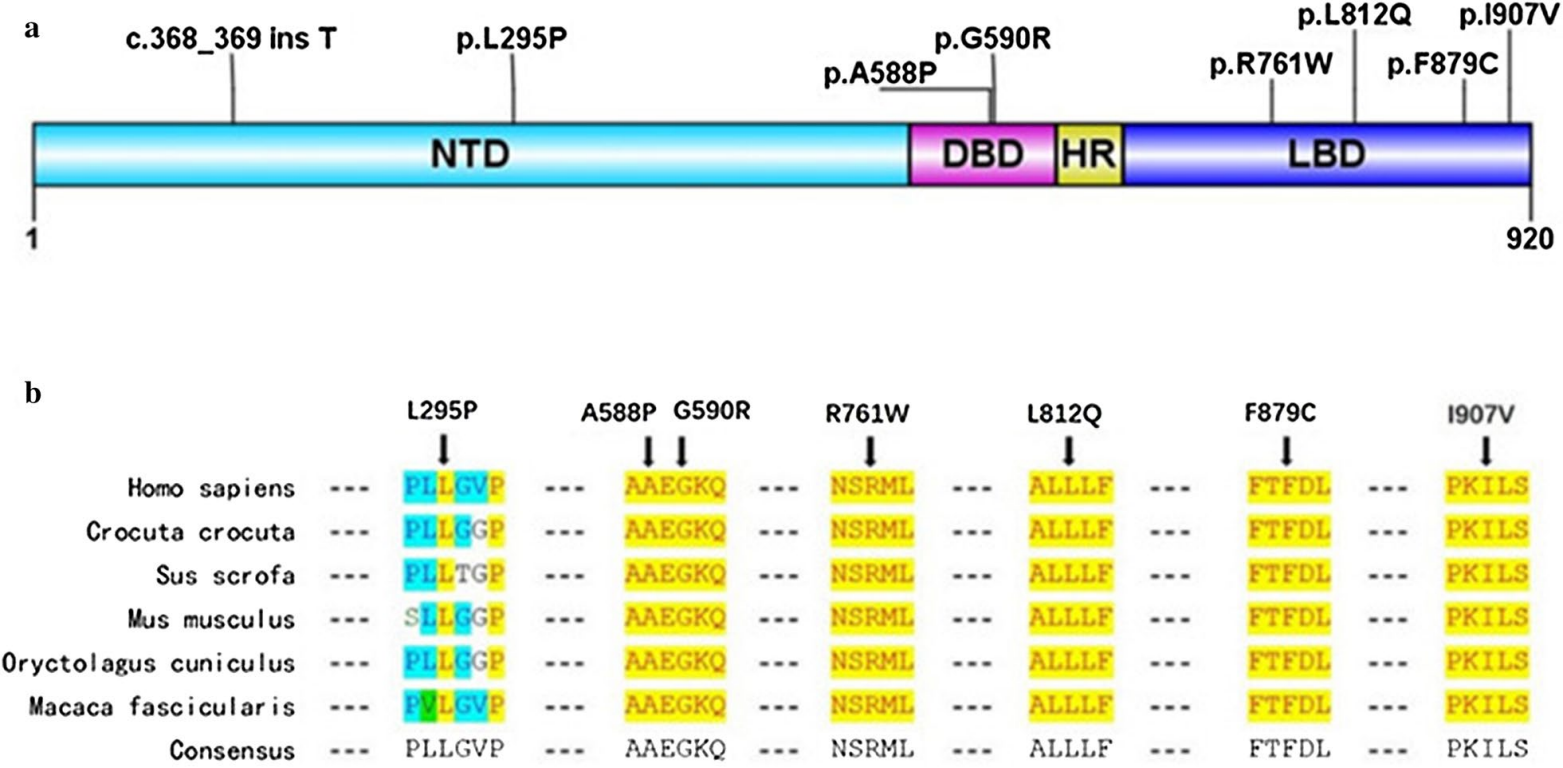
Distribution of eight novel *AR* mutations. **a** Novel *AR* mutations were distributed throughout the entire gene. **b** Amino acids L295, A588, G590, R761, L812, F879, and I907 are highly conserved through evolution. *NTD* N-terminal domain, *DBD* DNA-binding domain, *HR* hinge region, *LBD* ligand-binding domain

From the 150 subjects, a total of 23 variants of the *AR* gene were identified in 36 patients from 31 unrelated families (Table 1). Eight variants, including c.368_369 ins T (producing a truncated protein of 156 amino acids), p.L295P, p.A588P, p.G590R, p.R761W, p.L812Q, p.F879C and p.I907V, were novel (Table 1). Except for c.368_369 ins T, all the amino acid residues of novel variants were distributed throughout the entire gene and were highly conserved during evolution (Fig. 1). One PAIS patient exhibited the somatic mosaicism of an *AR* mutation. Seven unrelated patients (19.4%) harbored missense mutations at codon 841, with different amino acid changes (p.R841C or p.R841H). Patient 20 had 2 different missense *AR* mutations (p.P392R and p.R841C). Patient 36 was observed to have large deletion from exon 2 to exon 8 of *AR* by capture-based NGS. Moreover, 30.6% (11/36) of patients carried more than 2 variants other than *AR*, especially patient 3, who harbored variants in four other related genes (Table 1).

**Table 1:** Molecular basis of 36 Chinese 46, XY DSD patients and in-silico analyses of *AR* variants

No	cDNA	Protein	Functional domain	SIFT	Poly Phen2	Mutation taster	ACGM classification		Other gene mutations	Definite diagnosis
							Pre-functional assay	Post-functional assay		
1	c.2719 A>G	p.I907V^**a**^	LBD	T	B	DC	VUS	Likely pathogenic	N	PAIS
2	c.2719 A>G	p.I907V^**a**^	LBD						N	PAIS
3	c.2636T>G	p.F879C^**a**^	LBD	Dele	D	DC	VUS	Likely pathogenic	*DGKK* p.A106T(c.316 C>T); *EGF* p.I6V(c.16 A>G); *FKBP4* p.L356F(c.1066 C>T); *GJA4* p.R243W(c.727 C>T)	PAIS
4	c.2636T>G	p.F879C^**a**^	LBD						N	PAIS
5	c.1762 G>C	p.A588P^**a**^	DBD	Dele	D	DC	VUS	Likely pathogenic	N	CAIS
6	c.1762 G>C	p.A588P^**a**^	DBD						N	CAIS
7	c.1762 G>C	p.A588P^**a**^	DBD						N	CAIS
8	c.1762 G>C	p.A588P^**a**^	DBD						N	CAIS
9	c.2435T>A	p.L812Q^**a**^	LBD	Dele	D	DC	VUS	Likely pathogenic	N	CAIS
10	c.1768 G>C	p.G590R^**a**^^,^^**b**^	DBD	Dele	D	DC	VUS		*BMP4* p.R269Q(c.806 C>T); *POR* p.G626S(c.1876 G>A) *MAP3K1* p.V889L(c.2665 G>C)	PAIS
11	c.2281 A>T	p.R761W^**a**^	LBD	Dele	D	DC	VUS	Likely pathogenic	N	PAIS
12	c.368_369 ins T	c.368_369 ins T^**a**^					VUS	pathogenic	N	CAIS
13	c.884T>C	p.L295P^**a**^	NTD(Tau-1)	Dele	D	DC	VUS	Likely benign	*NR5A1* p.T29K(c.86 C>A); *MYH6* p.A1411T(c.4231 C>T)	NR5A1
14	c.1822 C>T	p.R608X				A	Pathogenic		N	CAIS
15	c.2667 C>T	p.S889S	LBD				Likely benign		*SRD5A2* p.V89L(c.264 G>C) (SNP)	PAIS
16	c.2667 C>T	p.S889S	LBD						N	PAIS
17	c.2521 C>T	p.R841C	LBD	Dele	D	A	Likely pathogenic		*CBX2* p.G185E(c.554 G>A)	PAIS
18	c.2521 C>T	p.R841C	LBD							PAIS
19	c.2521 C>T	p.R841C	LBD							PAIS
20	c.1175 C>G c.2521 C>T	p.P392R; p.R841C	NTD(Tau-5); LBD	Dele	D	DC	VUS		*ESR1* p.P146Q(c.437 C>A)	PAIS
21	c.2522 G>A	p.R841H	LBD	Dele	D	A	Likely pathogenic		N	CAIS
22	c.2522 G>A	p.R841H	LBD						N	PAIS
23	c.2522 G>A	p.R841H	LBD						N	PAIS
24	c.2437 C>T	p.L813F	LBD	T	B	DC	Likely pathogenic		N	PAIS
25	c.2437 C>T	p.L813F	LBD						N	PAIS
26	c.528 C>A	p.S176R	NTD(Tau-1)	Dele	D	DC	VUS		*POR* p.E119Q(c.357 G>C);*GJA4* p.R243W(c.727 C>T)	PAIS
27	c.528 C>A	p.S176R	NTD(Tau-1)						N	PAIS
28	c.528 C>A	p.S176R	NTD(Tau-1)						*SRD5A2* p.R227Q(c.680 G>A)	PAIS
29	c.2612 C>T	p.A871V	LBD	T	B	DC	Likely pathogenic		*BMP7* p.T251M(c.C752T)	PAIS
30	c.2057T>C	p.V686A	LBD	T	B	DC	VUS		N	PAIS
31	c.2227 A>G	p.M743V	LBD	Dele	P	DC	VUS		*PHF6* p.N147S(c.440A>G)	PAIS
32	c.2710 G>A	p.V904M	LBD	Dele	P	DC	VUS		N	CAIS
33	c.1846 C>G	p.R616G	DBD	Dele	D	DC	Likely pathogenic		N	PAIS
34	c.2104 C>T	p.L702F	LBD	Dele	D	DC	Likely pathogenic		N	CAIS
35	c.1739 G>T	p.C580F	DBD	Dele	D	A	Likely pathogenic		N	PAIS
36	exon 2–8 del	exon 2–8 del					pathogenic		*BMP2* p.R131S(c.A393T)	CAIS

*LBD* ligand-binding domain, *DBD* DNA-binding domain, *NTD* N-terminal domain, *Tau* transcription activation units, *SIFT* Sorting Intolerant From Tolerant, *T* tolerated, *Dele* deleterious, *B* benign, *D* probably damaging, *P* possibly damaging, *DC* disease causing, *A* disease causing automatic, *ACMG* American College of Medical Genetics and Genomics, *VUS* variant of uncertain significance, *PAIS* partial androgen insensitive syndrome, *CAIS* complete androgen insensitive syndrome, *NR5A1* nuclear receptor subfamily 5 Group A member 1

^a^Novel mutation

^b^Mosaic mutation

### Clinical features

The clinical features of the patients were presented in Additional file 1: Table S3. Thirty-six patients, aged 0.9 year to 62 years, presented various phenotypes of undermasculinization, such as microphallus or clitoromegaly, which was the most frequent symptom observed in this cohort.

According to the phenotype, AIS patients were divided into two subgroups, the CAIS (n = 11) and PAIS (n = 24) group. All the CAIS patients were reared as females. Sixteen patients in the PAIS group were raised as male, six were reared as females, and two patients underwent gender reassignment after puberty. Gynecomastia was found in 95% (21/22) of postpubertal patients with AIS. Patients 5 and 9 underwent ‘inguinal hernia surgery’(orchiectomy) before their first visit to our hospital. Thirteen patients had a family history of similar clinical presentation.

**Fig. 2:**
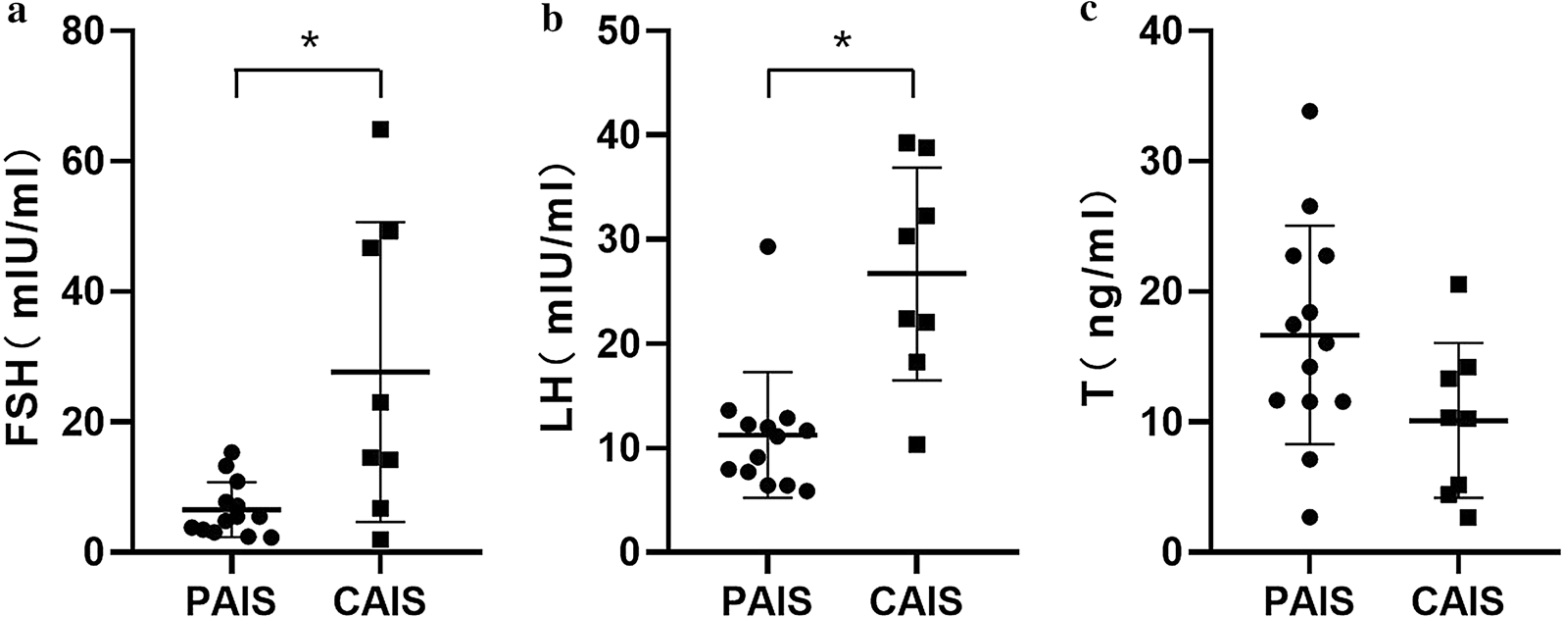
The scatterplot of the serum hormones for postpubertal patients with CAIS and PAIS except for patient 5, 9 and 12. **a** The scatterplot of follicle-stimulating hormone (FSH). **b** The scatterplot of luteinizing hormone (LH). **c** The scatterplot of testosterone (T). CAIS (n = 11); PAIS (n = 24); *P < 0.05

The serum levels of FSH, LH, T and AMH and the ASI are presented in Additional file 1: Table S3. FSH levels (18.8 [8.6, 48.7] vs. 5.5 [3.3, 9.3]; *P* < 0.05) and Serum LH (26.3 [19.2, 37.2] vs. 11.1 [7.1,12.6]; *P* < 0.01) were significantly higher and EMS was significantly lower (*P* < 0.05) in postpubertal patients with CAIS than those in patients with PAIS (Fig. 2a, b and Additional file 1: Table S4). However, no significant differences were observed in serum concentrations of T (10.3 [4.6, 14.0] vs. 16.1 [11.6, 22.8]; *P* > 0.05) or ASI (214.8 [105.3, 447.9] vs. 157.7 [90.1, 240.4]; *P* > 0.05) between the CAIS and PAIS groups (Fig. 2c and Additional file 1: Table S4).

### AR in vitro functional assays

**Fig. 3:**
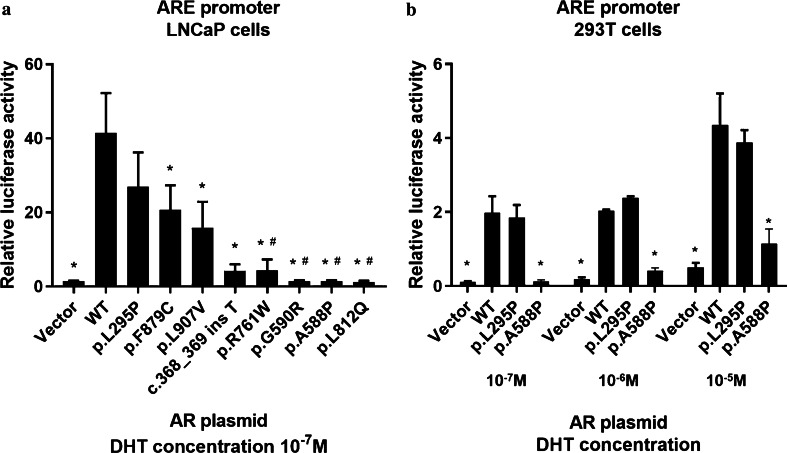
The transcriptional activities of WT and mutant AR were investigated using the androgen response element (ARE) promoter. The relative luminescence activity was expressed as a ratio of firefly to Renilla luciferase. **a** WT and all of the novel mutant AR in LNCaP cells. **b** WT AR, p.L295P, and p.A588P (as positive control) in 293T cells cultured with variant concentrations of DHT. The error bars represent the standard error. *versus WT *P* < 0.05; **#** versus Vector *P* > 0.05

After exploring the concentrations of  DHT,  we perfomed the assay with incubation  of 10^−7^ M DHT to obtain the adequate transcriptional activity of AR in vitro (Additional file 2: Supplemental Fig. 1).  All the novel mutant receptors exhibited markedly impaired transcriptional activity in the functional assays compared to that in cells transfected with wild-type AR (*P* < 0.05), except p.L295P (Fig. 3a). This mutation was detected in a 19-year-old patient whose clinical features were not similar to typical AIS. This patient showed a relatively developed penis (length of 4 cm), no gynecomastia, extremely elevated serum LH (51.33 mIU/ml) and FSH (79.94 mIU/ml) levels, relatively lower T (2.8 ng/ml, normal range 1.42–9.23 ng/ml) level and significantly reduced AMH level (0.44 ng/ml) (Additional file 1: Table S3). In the further assays in HEK 293T cells, mutant AR with p.L295P retained transcriptional activity similar to AR WT cultured with variant concentrations of DHT (Fig. 3b). Notably, the p.T29K mutation in the *NR5A1* gene and the p.A1411T mutation in the *MYH* gene were identified in this patient (Table 1). The transcriptional activities in LNCaP cells transfected with p.A588P, p.G590R, p.R761W and p.L812Q variants were significantly reduced, similar to those in cells transfected with empty vector (*P* > 0.05) (Fig. 3a).

**Fig. 4:**
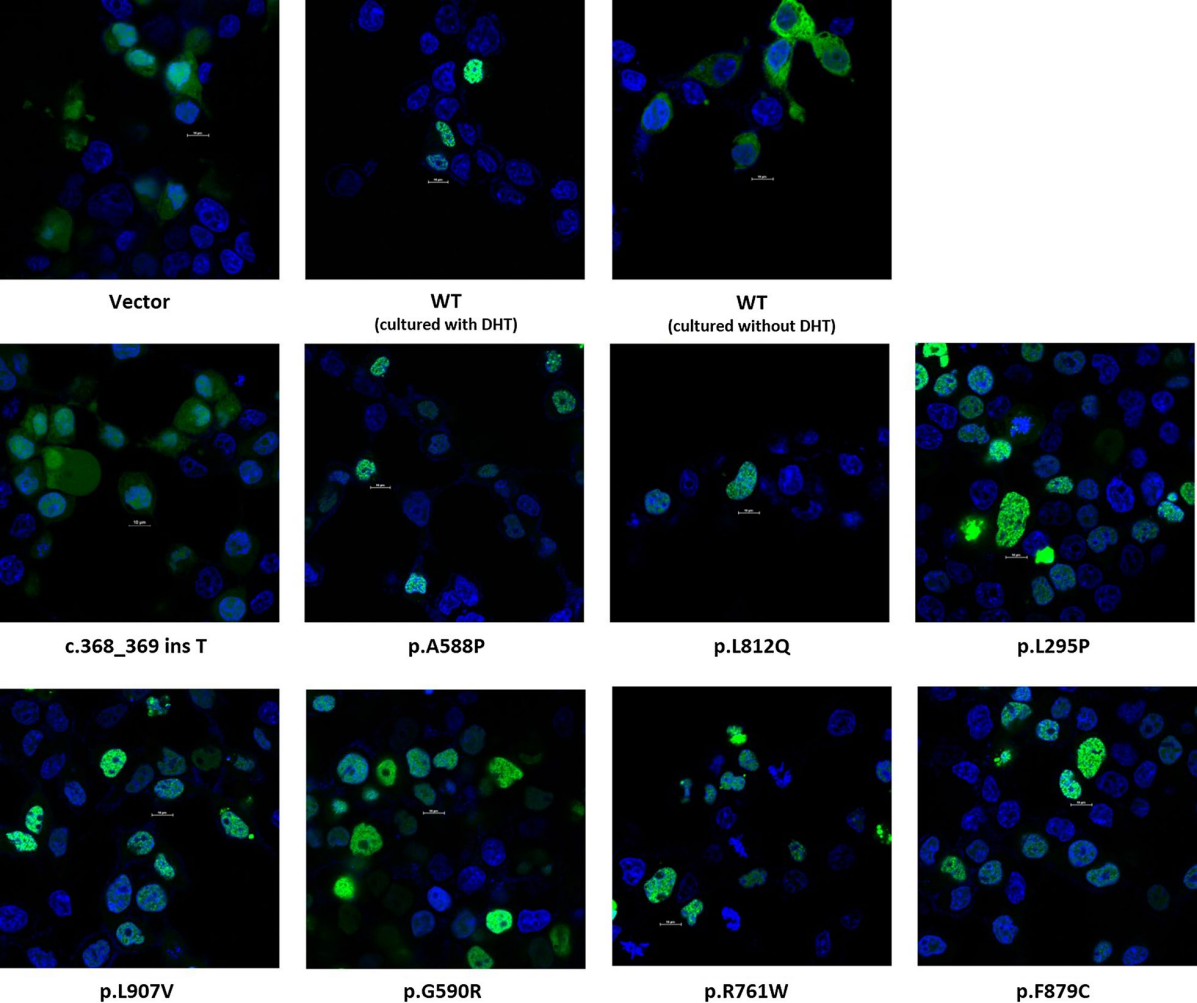
Nuclear translocation of GFP-tagged wild-type and mutant ARs. The upper panel from left to right: Empty vector and GFP-tagged WT AR (cultured without DHT) exhibited diffused localization in the cytoplasm. GFP-tagged WT AR (cultured with DHT) exhibited strong nuclear localization. The middle panel and lower pannel: Nuclear translocation of GFP-tagged eight mutant ARs in DHT treated cells. A similar nuclear localization pattern was observed in 7 novel missense mutants, while, the c.368_369 ins T mutant exhibited nuclear localization similar to empty vector. The scale bar represents 10 μm

GFP-tagged WT ARs showed nuclear localization with a uniform distribution. A similar nuclear localization pattern was observed for the seven novel missense mutants (p.L295P, p.A588P, p.G590R, p.R761W, p.L812Q, p.F879C and p.I907V). However, the AR mutant c.368_369 ins T showed a distinct cytoplasmic localization, which suggests impaired nuclear translocation (Fig. 4).

### Pathogenicity prediction of *AR* variants

All the novel *AR* variants were initially predicted to be VUS. However, according to the guideline of ACMG, based on the results of in vitro functional assays (PS3) and absence from controls in 1000 Genomes Project (PM2), all of the novel variants were reclassified as likely pathogenic except P.L295P. This variant met ‘Well-established in vitro functional studies show no damaging effect on protein function’ and ‘Variant found in a case with an alternate molecular basis for disease’ (BS3 and BP5) and was reclassified as likely benign, despite it was also predicted to be damaging by SIFT, Polyphen-2 and MutationTaster analysis (Table 1) [12]. Mutation c.368_369 ins T was reclassified as pathogenic according to ACMG guideline (PVS1 + PS3).

## Discussion

Due to similar clinical manifestations, it is difficult to distinguish AIS, especially PAIS, from other causes of 46, XY DSDs, such as partial gonadal dysgenesis and 5α-reductase 2 deficiency [7]. The typical serum hormone profile of pubertal AIS patients is characterized by an elevated or normal basal T level, an increased LH level, and a high ASI but a normal or mild elevated FSH level, which is also recommended for the selection of atypical subjects for analyses of the *AR* gene [15, 17, 18]. Melo et al. [18] found no significant differences in serum LH, FSH, DHT or T levels between patients with CAIS and PAIS. In the present study, we found that serum LH and FSH levels of the Chinese postpubertal patients with CAIS were significantly higher than those of postpubertal PAIS patients (*P* < 0.05) (Fig. 2).

A severe AIS phenotype may be related to LBD mutations, especially mutations between amino acid residues 688–712, 739–784 or 827–870, which comprise the greater part of the ligand-binding pocket [1]. Accordingly, 66.7% (6/9) of our patients harboring the mutations located in these regions presented with more severe phenotypes (EMS 2–3), except three patients (patients 17, 19 and 20, EMS 5.5–8) with p.R841C, which was previously found in male individuals with preserved fertility and reached an increased N/C interaction at higher T or various DHT concentrations in vitro [18, 19]. Herein, 19.4% of unrelated patients harbored *AR* mutations at the same residue (three with p.R841H and four with p.R841C), suggesting the Arg 841 residue is a mutation ‘hotspot’ in Chinese AIS patients.

The correlations between genotype and phenotype were not clarified in AIS patients [6, 11, 15, 20]. Oligogenicity, somatic mosaicism and AR coregulators (activators and repressors) may contribute to the variation of phenotypes [21–23]. In the present study, 30.6% of the patients had variants in more than 2 related genes using targeted NGS (Table 1). Notably, some of these sequenced genes were associated with human DSD, such as *SRD5A2* mutations causing 5ɑ-reductase type 2 deficiency and variants in the *CBX2* gene reported in prenatal 46, XY phenotypic females [24, 25]. However, it does not mean that patients with more mutations in different genes manifested more severe symptoms in this cohort, such as patient 3 (EMS = 6), who harbored variants in four other related genes. Although rarely described in AIS, somatic mosaicism may also contribute to the variation in AIS phenotypes [21]. In our study, four novel mutations exhibited a complete loss of androgen-induced transcriptional activity (Fig. 3a). In accordance with this result, six patients with p.L812Q, p.A588P or p.R761W exhibited a severe phenotype (EMS = 2). However, patient 10, containing p.G590R and three more variants in other related genes, presented a mild clinical feature (EMS = 7), in whom the somatic mosaicism of *AR* mutation was detected (Table 1 and Additional file 1: Table S3).

So far *AR* mutation has been considered as the confirmed cause for AIS, although it was not found in all patients clinically diagnosed with AIS [4]. The reduced *AR* mRNA expression in genital skin-derived fibroblasts, single-nucleotide polymorphisms (SNPs) paralleled by reduced *AR* expression, mutations outside the *AR* coding sequence, and variants in *AR* cofactors crucial for proper AR activity were also presumed potential causes [5, 13, 26]. Therefore, the impaired function of AR, rather than the *AR* mutations themselves, causes AIS. However, most of the reported *AR* mutations detected in DSD patients were predicted to be pathogenic or likely pathogenic via in silico tools instead of functional assays in vitro. In general, most algorithms are 65–80% accurate for missense variant prediction when examining known disease variants [12]. According to the guideline of ACMG, well-established in vitro functional studies (PS3 and BS3) may reclassify the variants. In a previous study, 61 *SCN5A* variants initially predicted as VUS by in-silico analyses were reclassified as benign/likely benign (n = 14), likely pathogenic (n = 35) and still VUS (n = 12) via high-throughput automated patch clamping [27]. In our study, it was found that all the novel *AR* variants, which were initially predicted to be VUS, significantly impaired the transcriptional activity of AR and were reclassified as pathogenic/likely pathogenic, except p.L295P (Table 1 and Fig. 3a). In rare cases, *AR* mutations have no influence on AR function in vitro, such as p.P392R (p.P390R using the former numbering system) [28], similar to p.L295P found in our patient 20, which is also located in an important region for transcriptional activity within the NTD [9, 10] and was predicted to be a VUS in-silico. This mutation was first reported in a CAIS patient with three different *AR* mutations (p.P392R, p.Q445R and p.E213E) [28]. However, p.P392R, neither independently nor in combination with p.Q445R, revealed any androgen-binding or transactivation abnormality in vitro [28]. Another mutation at the same amino acid residue (p.P392S) was reported in two unrelated individuals with oligospermia and did not lead to alterations of AR transcriptional activity in vitro, either [15]. In a CAIS patient with two *AR* mutations (p.Q799E and p.C807F), in vitro assays showed that these two mutations impaired AR function and exerted a synergistic negative effect on the AR transcriptional ability in combination [8, 15]. Unlike this patient, subject 18 exhibited a milder clinical phenotype (EMS = 7) despite harboring two different *AR* mutations (p.P392R and p.R841C), which was consistent with the results of the transcriptional assays of these two mutations in vitro.

To eliminate the possibility of potential interference, we repeated the transcriptional assays of p.L295P in HEK 293T cell system. Consistently, mutant ARs with p.L295P maintained normal transcriptional activity similar to the WT AR protein, even with various DHT concentrations (Fig. 3b). Previous studies showed that serum AMH was abnormally increased in CAIS children and remained at an extremely high level in postpubertal patients with CAIS because of the dysfunctional ARs in normal Sertoli cells [29]. However, patient 13 with p.L295P presented an obviously different serum hormone profile, including a relatively lower T level, an extremely elevated serum FSH level and a drastically reduced AMH level (Additional file 1: Table S3). Gynecomastia, which is one of the indicators of AIS, even MAIS [17], was also absent from this patient. We revealed two additional variants in this patient, p.T29K of *NR5A1* and p.A1411T of *MYH*, by targeted NGS (Table 1). The *NR5A1* gene encodes steroidogenic factor 1, which is essential for sexual differentiation and formation of the primary steroidogenic tissues [30]. Our previous study found that p.T29K mutation altered subnuclear structure and impaired the transactivation ability of NR5A1 in vitro [3]. Therefore, all the clinical features of patient 13, including the extremely reduced AMH level, the lack of gynecomastia and a comparatively developed penis, especially under the effects of a relatively low T concentration, may be explained by the dysfunction of the *NR5A1* gene resulting from the mutation p.T29K and suggested the improper diagnosis of PAIS in this patient. *AR* variant p.L295P was also reclassified as likely benign due to the criteria BS3 and BP5.

In conclusion, 23 *AR* variants were identified in 36 Chinese 46, XY DSD patients by using targeted NGS. Out of eight novel variants initially predicted as VUS, seven were reclassified as pathogenic/likely pathogenic and one was reclassified as likely benign due to the results of in vitro functional assays. In Chinese patients with AIS, amino acid residue Arg841 was one of the mutation hotspots in the *AR* gene, and the higher LH and FSH levels may be indicative of CAIS postpuberty. *AR* variants may not significantly impair AR function in some cases, which suggests the importance of functional assays, instead of in-silico analyses, especially in patients with multiple gene variants.

## Supplementary Information

**Additional file 1**. Supplemental tables for related genes, primers and clinical data.

**Additional file 2: Supplemental Fig. 1**. The influence of DHT concentration in AR functional assays in vitro. The transcriptional assay was investigated with WT AR plasmid and androgen response element (ARE) promoter. The transfected LNCap cells were incubated with 0.1% dimethyl sulfoxide (DMSO, as negative control) or variable concentrations of DHT. The relative luminescence activity was expressed as a ratio of firefly to Renilla luciferase. The error bars represent the standard error. *, vs 0.1% DMSO *P* < 0.05; #, vs 10^−7^ M DHT *P* > 0.05

## Data Availability

The datasets used and/or analysed during the current study are available from the corresponding author on reasonable request.

## Abbreviations

AIS: Androgen insensitive syndrome
AR: Androgen receptor
DSD: Disorder/differences of sex development
NGS: Next-generation sequencing
CAIS: Complete androgen insensitive syndrome
PAIS: Partial androgen insensitive syndrome
MAIS: Mild androgen insensitivity syndrome
T: Testosterone
DHT: Dihydrotestosterone
NTD: N-terminal domain
DBD: DNA-binding domain
LBD: Ligand-binding domain
Tau: Transcription activation units
APOD: Apolipoprotein D
SNPs: Single-nucleotide polymorphisms
ACMG: American College of Medical Genetics and Genomics
VUS: Variants of uncertain clinical significance
EMS: External masculinization score
LH: Luteinizing hormone
FSH: Follicle-stimulating hormone
AMH: Anti-Mullerian hormone
ASI: Androgen sensitivity index
ARE: Androgen-responsive element
WT: Wide-type
HEK: Human embryonic kidney
