# Supplementary material for: Phenotypic and biochemical characteristics and molecular basis in 36 Chinese patients with androgen receptor variants
Source: Orphanet J Rare Dis. 2021 Mar 9;16:122. doi: 10.1186/s13023-021-01765-w (PMC7942007; doi:10.1186/s13023-021-01765-w)
Supplement: Supplementary file 1 — Additional file 1. Supplemental tables for related genes, primers and clinical data. [file 13023_2021_1765_MOESM1_ESM.docx]

**Supplemental Table 1. 80 genes included in targeted next-generation sequencing**

| **Genes associated with gonad development** | | | | | | | |
| --- | --- | --- | --- | --- | --- | --- | --- |
| NR5A1 | nuclear receptor subfamily 5, group A, member 1 | SRY | sex determining region Y | RSPO1 | R-spondin 1 | SOX9 | SRY (sex determining region Y)-box 9 |
| WT1 | Wilms tumor 1 | NR0B1 | nuclear receptor subfamily 0, group B, member 1 | WNT4 | wingless-type MMTV integration site family, member 4 | MAP3K1 | mitogen-activated protein kinase kinase kinase 1, E3 ubiquitin protein ligase |
| SOX3 | SRY (sex determining region Y)-box 3 | GATA4 | GATA binding protein 4 | CBX2 | chromobox homolog 2 | TESC | tescalcin |
| CST9 | cystatin 9 | CTNNB1 | catenin (cadherin-associated protein), beta 1 | FST | follistatin | BMP2 | bone morphogenetic protein 2 |
| BMP15 | bone morphogenetic protein 15 | BMP4 | bone morphogenetic protein 4 | BMP7 | bone morphogenetic protein 7 | DHH | Desert Hedgehog |
| SHOX | PHOG/short stature homeobox | DMRT1 | double sex, Mab3–related transcription factor 1 | SOX8 | SRY (sex determining region Y)-box 8 | TSPYL1 | TSPY-like 1 |
| GJA4 | gap junction protein, alpha 4 | SHH | sonic hedgehog | WNT5A | Wnt family member 5A | BMPR1A | bone morphogenetic protein receptor type 1A |
| MAMLD1 | mastermind-like domain–containing 1 | ARX | aristaless-related homeobox, X-linked gene | ARNT2 | aryl hydrocarbon receptor nuclear translocator 2 | FGF8 | fibroblast growth factor 8 |
| FGF10 | fibroblast growth factor 10 | FGFR2 | fibroblast growth factor receptor 2 | AMHR2 | anti-Mullerian hormone receptor, type II | AMH | anti-Mullerian hormone |
| LHX9 | LIM homeobox 9 |  |  |  |  |  |  |

**Supplemental Table 1. 80 genes included in targeted next-generation sequencing**

| **Genes associated with sex differentiation** | | | | | | | |
| --- | --- | --- | --- | --- | --- | --- | --- |
| STAR | steroidogenic acute regulatory protein | LHCGR | luteinizing hormone/ choriogonadotropin receptor | CYP11A1 | cytochrome P450, family 11, subfamily A, polypeptide 1 | CYP17A1 | cytochrome P450, family 17, subfamily A, polypeptide 1 |
| HSD3B2 | hydroxy-delta-5-steroid dehydrogenase, 3 beta- and steroid delta-isomerase 2 | HSD11B1 | hydroxysteroid 11-beta dehydrogenase 1 | HSD17B3 | hydroxysteroid (17-beta) dehydrogenase 3 | SRD5A2 | steroid-5-alpha-reductase, alpha polypeptide2 |
| AR | androgen receptor | POR | P450 (cytochrome) oxidoreductase | CYB5A | cytochrome b5 type A (microsomal) | LHB | luteinizing hormone beta polypeptide |
| FDX1 | ferredoxin 1 | FDXR | ferredoxin reductase | FKBP4 | FK506 binding protein 4 | PIP | prolactin induced protein |
| FOXL2 | forkhead box L2 | PHF6 | PHD finger protein 6 |  |  |  |  |
| **Other genes associated with hypogonadism, hypospadias and certain clinical syndrome** | | | | | | | |
| MAP3K11 | mitogen-activated protein kinase kinase kinase 11 | LHX1 | LIM homeobox 1 | EMX2 | empty spiracles homeobox 2 | KIAA1310 | KAT8 regulatory NSL complex subunit 3 |
| MID1 | midline 1 | RXFP2 | relaxin/insulin like family peptide receptor 2 | PTCH1 | patched 1 | HOXA13 | homeobox A13 |
| ATF3 | activating transcription factor 3 | HOXD13 | homeobox D13 | MSX1 | msh homeobox 1 | EPHB2 | EPH receptor B2 |
| MAFB | MAF bZIP transcription factor B | MYH6 | myosin heavy chain 6 | CYR61 | cysteine rich angiogenic inducer 61 | CTGF | connective tissue growth factor |
| GADD45A | growth arrest and DNA damage inducible alpha | DGKK | diacylglycerol kinase kappa | GSTM1 | glutathione S-transferase mu 1 | EGF | epidermal growth factor |
| HOXA4 | homeobox A4 | HOXB6 | homeobox B6 | ESR1 | estrogen receptor 1 | ESR2 | estrogen receptor 2 |
| BNC2 | basonuclin 2 |  |  |  |  |  |  |

**Supplemental Table 2. Primers for plasmid construction**

| **Plasmid template** | **Target plasmid/ mutation** | **Forward** | **Reverse** |
| --- | --- | --- | --- |
| pCMV-hAR-WT | pEGFP-N2-AR-WT | 5’CCGCTCGAGATGGAAGTGCAGTTAGGGCTG 3’ | 5’CGCGGATCCACTGGGTGTGGAAATAGATGGG 3' |
| pCMV-hAR-WT/  pEGFP-N2-AR-WT | p.L812Q | 5’TGTGCATGAAAGCACTGCAACTCTTCAGCA 3’ | 5’TGCAGTGCTTTCATGCACAGGAATTCCT 3’ |
| pCMV-hAR-WT/  pEGFP-N2-AR-WT | p.I907V | 5’CTGTGCAAGTGCCCAAGGTCCTTTCTGGG 3’ | 5’CCTTGGGCACTTGCACAGAGATGATCTC 3’ |
| pCMV-hAR-WT/  pEGFP-N2-AR-WT | p.A588P | 5’GTCTTCTTCAAAAGAGCCCCTGAAGGGAAA 3’ | 5’GGGCTCTTTTGAAGAAGACCTTGCAGC 3’ |
| pCMV-hAR-WT/  pEGFP-N2-AR-WT | c.368_369 insT | 5’CGCAGTCGGCCCTTGGAGTGCCAC 3' | 5’AAGGGCCGACTGCGGCTGTGAAGG 3' |
| pCMV-hAR-WT/  pEGFP-N2-AR-WT | p.G590R | 5’CTTCAAAAGAGCCGCTGAACGGAAACAGAAGT 3’ | 5’GTTCAGCGGCTCTTTTGAAGAAGACCTTG 3’ |
| pCMV-hAR-WT/  pEGFP-N2-AR-WT | p.F879C | 5’AGCTGCATCAGTTCACTTGTGACCTGCTAA 3’ | 5’CAAGTGAACTGATGCAGCTCTCTCGCAATAG 3’ |
| pCMV-hAR-WT/  pEGFP-N2-AR-WT | p.R761W | 5’TTCACCAATGTCAACTCCTGGATGCTCTAC 3’ | 5’AGGAGTTGACATTGGTGAAGGATCGCCAGC 3’ |
| pCMV-hAR-WT/  pEGFP-N2-AR-WT | p.L295P | 5’GAATGCAAAGGTTCTCTGCCAGACGACAGCG 3’ | 5’GGCAGAGAACCTTTGCATTCGGCCAATGGGG 3 |

The underlined is the restriction enzyme sites. Xho I: CTCGAG; BamH I: GGATCC

**Supplemental Table 3 Clinical features and serum hormone of 36 Chinese 46, XY DSD patients**

| **No.** | **Ages** | **Social Gender** | **Clinical**  **Features** | **Gynecomastia** | **Surgical Operations** | **Family History** | **EMS** | **FSH**  **(mIU/ml)** | **LH**  **(mIU/ml)** | **T**  **(ng/ml)** | **ASI** | **AMH**  **(ng/ml)** | **SHBG**  **(nmol/L)** |
| --- | --- | --- | --- | --- | --- | --- | --- | --- | --- | --- | --- | --- | --- |
| 1 | 26 | F to M | MP，H，BS，UC | Y | 1,5 | Y^a^ | 2.5 | 7.17 | 13.63 | 11.57 | 157.70 | 8.90 | 60.34 |
| 2 | 23 | F to M | MP，H，BS，BC | Y | 1,5 | Y^a^ | 2 | 15.35 | 12.9 | 18.43 | 237.75 | 14.09 | 111.2 |
| 3 | 31 | M | MP，H | Y | 1,3,5 | N | 6 | 10.89 | 11.13 | 16.09 | 179.08 | UD | 63.73 |
| 4 | 3 | F | MP，H，BS，BC | underage | N | Y | 2 | 2.26 | 0.39 | 0.13 | 0.05 | UD | UD |
| 5 | 28 | F | MP，H，BS，BC | N | 2 | Y^b^ | 2 | 34.04 | 13.33 | 0.65 | 8.66 | UD | UD |
| 6 | 49 | F | MP，H，BS，BC | Y | N | Y^b^ | 2 | 46.71 | 22.38 | 5.18 | 115.93 | UD | UD |
| 7 | 54 | F | MP，H，BS，BC | Y | N | Y^b^ | 2 | 49.31 | 30.31 | 10.35 | 313.71 | UD | UD |
| 8 | 62 | F | MP，H，BS，BC | Y | N | Y^b^ | 2 | 64.88 | 39.28 | 2.67 | 104.88 | UD | UD |
| 9 | 16 | F | MP，H，BS，BC | N | 2 | N | 2 | 66.31 | 26.13 | 0.22 | 5.75 | 0.18 | UD |
| 10 | 27 | M | MP，H | Y | 1,4,5 | N | 7 | 13.32 | 12.28 | 7.15 | 87.80 | 7.67 | UD |
| 11 | 12 | M | MP，H，BS，BC | Y | N | N | 2 | 2.46 | 5.91 | 2.72 | 16.08 | UD | 14.88 |
| 12 | 25 | F | MP，H，BS，BC | Y | N | Y |  | 9.74**^†^** | 53.5**^†^** | 711.5**^†^** | 38065.25**^†^** | UD | UD |
| 13 | 19 | F to M | MP，H，BS，BC | N | 1,3,4 | N | 2.5 | 79.94 | 51.33 | 2.8 | 143.72 | 0.44 | 28.08 |
| 14 | 20 | F | MP，H，BS，BC | Y | 2 | N | 2 | 14.55 | 18.27 | 4.46 | 81.48 | UD | UD |
| 15 | 11 | M | MP，H，BS | underage | 1 | Y | 2 | 3.24 | 3.56 | 4.75 | 16.91 | 110.60 | UD |
| 16 | 18 | F | MP，H，BS，BC | Y | N | N | 2 | 7.79 | 29.33 | 11.69 | 342.87 | UD | 37.25 |
| 17 | 20 | M | MP，H，UC | Y | 1,3 | Y | 5.5 | 2.3 | 9.16 | 26.54 | 243.11 | 8.29 | 83.91 |
| 18 | 17 | M | MP，H，BS，UC | Y | N | Y | 2 | 5.49 | 6.43 | 22.75 | 146.28 | UD | UD |
| 19 | 19 | M | MP，H | N | 1 | N | 8 | 3.12 | 12.03 | 33.86 | 407.34 | UD | 65.95 |
| 20 | 8 | M | MP，H | underage | 4 | N | 7 | 1.52 | 0.13 | 0.13 | 0.02 | 84.40 |  |
| 21 | 20 | F | MP，H，BS，BC | Y | 2 | N | 2 | 6.78 | 10.36 | 10.29 | 106.60 |  |  |
| 22 | 15 | M | MP，H，BS，BC | Y | 3,4 | N | 2 | 3.49 | 7.98 | 11.57 | 92.33 | 12.04 |  |

**Supplemental Table 3 Clinical features and serum hormone of 36 Chinese 46, XY DSD patients with *AR* varants**

| **No.** | **Ages** | **Social Gender** | **Clinical**  **Features** | **Gynecomastia** | **Surgical Operations** | **Family History** | **EMS** | **FSH**  **(mIU/ml)** | **LH**  **(mIU/ml)** | **T**  **(ng/ml)** | **ASI** | **AMH**  **(ng/ml)** | **SHBG**  **(nmol/L)** |
| --- | --- | --- | --- | --- | --- | --- | --- | --- | --- | --- | --- | --- | --- |
| 23 | 26 | F | MP，H，BS，BC | Y | 2 | N | 2.5 | 3.86 | 7.73 | 17.49 | 135.20 | UD | 76.63 |
| 24 | 5 | F | MP，H，BS，BC | underage | 3 | Y^c^ | 2 | 3.71 | 0.33 | 0.13 | 0.04 | UD | UD |
| 25 | 0.9 | M | MP | underage | N | Y^c^ | 9 | 2.19 | 2.76 | 2.06 | 6.04 | UD | UD |
| 26 | 4 | M | MP，H，UC | underage | 1,3,4 | N | 6.5 | 2.17 | 0.56 | 0.13 | 0.07 | 97.30 | UD |
| 27 | 10 | M | MP，H，BC | underage | N | N | 5 | 7.47 | 3.33 | 2.19 | 7.29 | 14.83 | UD |
| 28 | 19 | M | MP | Y | N | N | 9 | 5.49 | 6.43 | 22.75 | 14.23 | UD | 11.51 |
| 29 | 5 | M | MP，H | underage | N | N | 7 | 1.08 | 0.08 | 0.13 | 0.01 | UD | 151.6 |
| 30 | 21 | M | MP，H | Y | 1 | N | 7 | 4.82 | 11.67 | 14.26 | 166.41 | UD | 50.11 |
| 31 | 5 | F | MP，H，BS，UC | underage | 3 | N | 2.5 | 1.49 | 0.33 | 0.13 | 0.04 | UD | >200.00 |
| 32 | 24 | F | MP，H，BS，BC | Y | N | N | 2 | 23.01 | 32.27 | 13.35 | 430.80 | 19.40 | 126 |
| 33 | 2 | M | MP，H | underage | 1 | N | 8 | 0.67 | 0.12 | 0.13. | 0.02 | 357.00 | 192.7 |
| 34 | 17 | F | MP，H，BS，BC | Y | 2 | N | 2 | 14.24 | 38.83 | 14.2 | 551.39 | UD | UD |
| 35 | 1 | F | MP，H，BS，UC | underage | N | N | 2.5 | 0.13 | 3.03 | 2.2 | 6.67 | UD | >200.0 |
| 36 | 20 | F | MP，H，BS，BC | Y | 2,4 | N | 2 | 1.96 | 22.06 | 20.56 | 453.55 | UD | 47.25 |

**Ages,** age at first visit; **M,** male; **F,** female; **MP,** micropenis; **H,** hypospadias; **BS,** bifid scrotum; **UC,** unilateral cryptorchid; **BC,** bilateral cryptorchid; **N,** no; **Y,** yes; **Surgical Operation:** **1,** surgical repair of hypospadias; **2,** orchiectomy; **3,** orchidorrhaphy; **4,** penis reconstruction; **5,** mastectomy; **EMS,** external masculinization scores; **FSH,** follicle-stimulating hormone; **LH,** luteinizing hormone; **T,** testosterone; **ASI,** androgen sensitivity index; **AMH,** [anti-Mullerian hormone](javascript:;); **SHBG,** sex hormone-binding globulin; **UD,** undetected;

**†**, patient 12 whose hormones were tested in another hospital with different methods and normal ranges

**a, b, c,** patients from the same family

**Supplemental Table 4 Comparison of FSH, LH, T, ASI, and EMS between postpubertal patients with CAIS and PAIS**

|  | **CAIS^†^** | **PAIS** | ***P* Value** |
| --- | --- | --- | --- |
| **Case Number** | 8 | 13 |  |
| **Ages** | 22.0 [20.0, 52.8] | 20.0 [17.5, 26.0] | > 0.05 |
| **FSH（mIU/ml）** | 18.8 [8.6, 48.7] | 5.5 [3.3, 9.3] | < 0.05 |
| **LH（mIU/ml）** | 26.3 [19.2, 37.2] | 11.1 [7.1,12.6] | < 0.01 |
| **T（ng/ml）** | 10.3 [4.6, 14.0] | 16.1 [11.6, 22.8] | > 0.05 |
| **ASI** | 214.8 [105.3, 447.9] | 157.7 [90.1, 240.4] | > 0.05 |
| **EMS** | 2.0 [2.0, 2.0] | 2.5 [2.0, 7.0] | < 0.05 |

**CAIS,** complete androgen insensitive syndrome; **PAIS,** partial androgen insensitive syndrome; **FSH,** follicle-stimulating hormone; **LH,** luteinizing hormone; **T,** testosterone; **ASI,** androgen sensitivity index; **EMS,** external masculinization scores; **†,** excluding patient 12 whose hormones were tested in another hospital with different methods and normal ranges and patients 5 and 9 who had undergone orchiectomy in prepuberty
