# Supplementary figures and images for: Phenotypic and biochemical characteristics and molecular basis in 36 Chinese patients with androgen receptor variants
Source: Orphanet J Rare Dis. 2021 Mar 9;16:122. doi: 10.1186/s13023-021-01765-w (PMC7942007; doi:10.1186/s13023-021-01765-w)

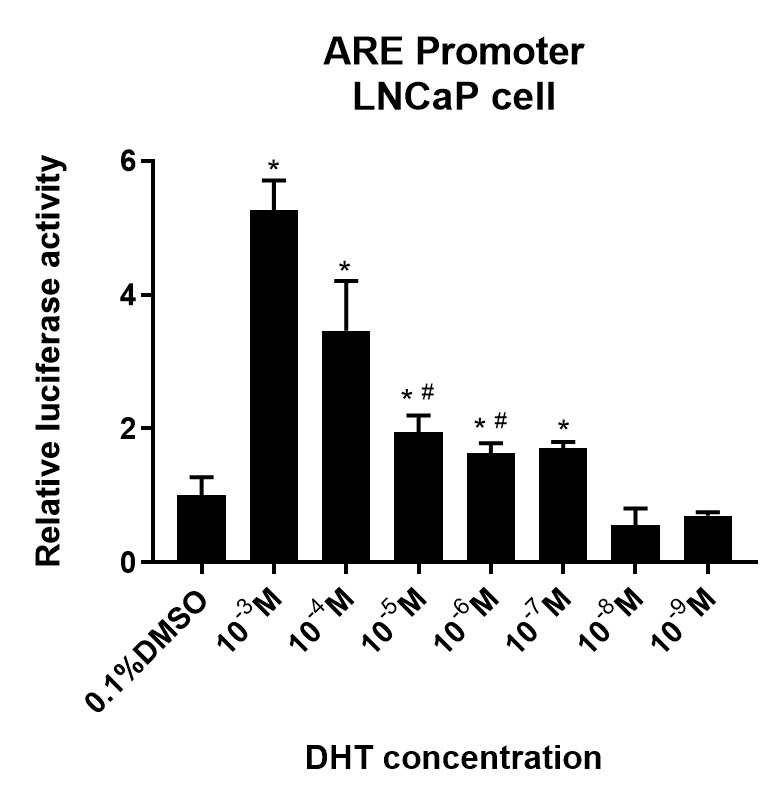

Supplement: Supplementary file 2 — Additional file 2: Supplemental Fig. 1. The influence of DHT concentration in AR functional assays in vitro. The transcriptional assay was investigated with WT AR plasmid and androgen response element (ARE) promoter. The transfected LNCap cells were incubated with 0.1% dimethyl sulfoxide (DMSO, as negative control) or variable concentrations of DHT. The relative luminescence activity was expressed as a ratio of firefly to Renilla luciferase. The error bars represent the standard error. *, vs 0.1% DMSO P < 0.05; #, vs 10−7 M DHT P > 0.05 [file 13023_2021_1765_MOESM2_ESM.jpg]
